# Designing a tool ensuring older patients the right medication at the right time after discharge from hospital– the first step in a participatory design process

**DOI:** 10.1186/s12913-024-10992-3

**Published:** 2024-04-24

**Authors:** Thorbjørn Hougaard Mikkelsen, Jens Søndergaard, Niels Kristian Kjær, Jesper Bo Nielsen, Jesper Ryg, Lene Juel Kjeldsen, Christian Backer Mogensen

**Affiliations:** 1 Emergency Department, Hospital Sønderjylland, Aabenraa, Denmark; 2 Research Unit of Emergency Medicine, Department of Regional Health Research, University of Southern Denmark, Odense, Denmark; 3 Research Unit of General Practice, Department of Public Health, University of Southern Denmark, Odense, Denmark; 4 Department of Clinical Research, University of Southern Denmark, Odense, Denmark; 5 Department of Geriatric Medicine, Odense University Hospital, Odense, Denmark; 6 The hospital pharmacy research unit, Hospital Sønderjylland, Aabenraa, Denmark

**Keywords:** Polypharmacy, Older people, Adverse drug reactions, Medication errors, Continuity of care, Participatory design, Qualitative research

## Abstract

**Background:**

On average, older patients use five or more medications daily, increasing the risk of adverse drug reactions, interactions, or medication errors. Healthcare sector transitions increase the risk of information loss, misunderstandings, unclear treatment responsibilities, and medication errors. Therefore, it is crucial to identify possible solutions to decrease these risks. Patients, relatives, and healthcare professionals were asked to design the solution they need.

**Methods:**

We conducted a participatory design approach to collect information from patients, relatives, and healthcare professionals. The informants were asked to design their take on a tool ensuring that patients received the correct medication after discharge from the hospital. We included two patients using five or more medications daily, one relative, three general practitioners, four nurses from different healthcare sectors, two hospital physicians, and three pharmacists.

**Results:**

The patients’ solution was a physical location providing a medication overview, including side effects and interactions. Healthcare professionals suggested different solutions, including targeted and timely information that provided an overview of the patient’s diagnoses, treatment and medication. The common themes identified across all sub-groups were: (1) Overview of medications, side effects, and diagnoses, (2) Sharing knowledge among healthcare professionals, (3) Timely discharge letters, (4) Does the shared medication record and existing communication platforms provide relevant information to the patient or healthcare professional?

**Conclusion:**

All study participants describe the need for a more concise, relevant overview of information. This study describes elements for further elaboration in future participatory design processes aimed at creating a tool to ensure older patients receive the correct medication at the correct time.

**Supplementary Information:**

The online version contains supplementary material available at 10.1186/s12913-024-10992-3.

## Background

Healthcare sector transitions increase the risk of information loss, misunderstandings, unclear treatment responsibilities, and medication errors [1–3]. Medication of older patients following hospital visits is often seen as particularly complex [4]. Polypharmacy adds significantly to this complexity due to the uncertainty about how often and for how long medication is needed, challenges in sharing information in sector transitions with different healthcare professionals, and the patients’ and relatives’ cognitive ability and motivation to follow medication plans [5]. During hospitalisation, 60% of patients receive three or more changes to their medication, and the risk of a harmful event increases significantly with each prescription change [6, 7]. Older patients often use five or more prescription medications daily [8, 9], but polypharmacy is not always beneficial for the patient [10–15], and some older patients experience severe side effects [16–20] often due to drug-drug interactions [21, 22]. In addition, previous parts of this study have shown that older patients are often concerned about drug-drug interactions and side effects as well as confused about aspects such as names, labels, and when to take the medication [23]. Therefore, the discharge of elderly patients from the hospital is a complex process where robust tools are needed to support the correct medication at the correct time. For international readers it is important to know a particular artefact in the Danish healthcare system. When the shared Medication Record (SMR) was established to document prescribed medications for a patient over ten years, a new word, “ordineret medicin,” was introduced, which translated means non-prescription medication. This phrase was introduced to distinguish between an active prescription and a passive non-prescription medication. The SMR is a continuously updated and accessible online overview for patients and healthcare professionals regardless of sector, and gives healthcare professionals, and patients access to view current medications, including dose and prescription redemption [24, 25]. SMR also enables healthcare professionals to see the patient’s medication history and register changes [26]. Upon discharge, GPs receive a discharge summary from the hospital describing the treatment and suggesting follow-up. If home care is needed, the municipality receives a patient treatment- and care plan from the hospital so the municipality can prepare for the patient’s return home. The patient treatment- and care plan will among other things include information regarding the hospitalization, diagnoses, medication, and required nursing and homecare support after discharge [27]. This knowledge is important to understand some of the results of this study. Despite these systems enabling sharing of information improvements are needed to ensure the right medication for older patients [23].

To develop a solution for solving major medication challenges facing polypharmacy patients when discharged from the hospital, we invited relevant actors to design their vision of the most suitable and robust tool. In this study, we will explore the first step in this design process of a future hopefully robust tool to be used, when patients cross healthcare sectors. Previous studies were typically based on input from only one stakeholder, whereas our study invited both clinicians from both healthcare sectors and patients into the same participatory design process with the purpose of developing a tool to be shared, appreciated and seen as useful for all stakeholders.

### Aim

This study aims to provide knowledge about key elements a future solution should include to ensure correct medication treatment for older patients transitioning between secondary and primary healthcare sectors.

## Methods

### Participatory design

This study focuses on older patients treated with five or more medications and we used a participatory design (PD) process including patients, relatives and healthcare professionals. PD is beneficial when exploring informants’ wishes and creating new solutions [28].

PD studies combine the use of different methods and activities running simultaneously during the entire process: Literature studies, field studies, design and development, and testing [29]. Within the health sciences, PD is typically broadly divided into 3 phases. In phase 1, the users’ needs are identified and discussed in this study using FGIs. In phase 2, a prototype is developed and designed through workshops. Mock-ups and proposed solutions are designed, tested, and retested to develop a prototype that can be pilot-tested [29–31]. In phase 3, the prototype is tested [29, 30]. This study reports the first step in phase 2 aiming at laying the foundation for developing prototypes and Mock-ups in future studies if financed.

We asked participants to create a tool or solution, to enhance adherence. We let the participants think, discuss, and report their reflections on the best solution [28, 32].

Our object was not defined a priori. Hence, we began the process with a brainstorm, where the participants were asked to list essential aspects to ensure all patients received the correct medication. The factors identified from the brainstorm were discussed in smaller groups of peer participants. The small groups were asked to design a solution, later presenting to the group how it would work [28, 32].

Brainstorming generates ideas emphasizing many solutions without consideration for practicalities [33, 34]. The participants were instructed not to be critical of ideas but to describe any additional ideas that come to mind, no matter how wild. It was emphasized that the brainstorm aimed to generate many ideas, and participants were encouraged to be innovative after hearing other’s ideas [34]. Many studies described brainstorming in groups as suboptimal in productivity compared to brainstorming individually beforehand [34–36]. Hence, individual brainstorming was conducted before participants shared ideas and initiated the design process.

### Setting and participants

Participant recruitment aimed at achieving rich and diverse perspectives [29]. GPs and nurses were invited to the participatory design process through one of the co-authors (NK) professional network and GPs associated with Hospital Sønderjylland, University Hospital of Southern Denmark. Homecare nurses were invited through their local municipality and hospital nurses through their departments. Pharmacists were invited through a local pharmacist. Patients and relatives were invited following admission to the emergency department if 72 years or older and managing five or more medications themselves or with the help of a relative and able to transport themselves to the PD process at the hospital. Patients with dementia were excluded. The participating patients and relatives have previously participated in focus group interviews (FGI) reported elsewhere [23] and were subsequently invited to participate in the participatory design process. The inclusion of patients invited to the FGIs was based on consecutive sampling among patients admitted to the Emergency Department at Hospital Sønderjylland. The patients were invited while admitted to the department during ten days in April, May and June 2021 [23]. Overall 31 patients were eligible for the FGIs. A total of 10 patients, here of three with a spouse, accepted the invitation to the FGIs. One died before the FGI and another did not show up [23]. Patients and relatives participating in the FGIs were invited to participate in the making process. The three pharmacists could not attend the participatory design process on the same day as the other actors and were invited to participate on an alternative day. All participants, except one hospital physician and one pharmacist, were Danish by ethnicity (ethnicity not stated due to anonymity aspects).

Six following groups of similar participants were created: (1) Three GPs, (2) Two chief physicians, (3) Three pharmacists, (4) Two nurses employed in general practice, (5) One participating hospital nurse was grouped with the two homecare nurses. (6) Two patients aged 73 and 78 years and one relative. In total, 16 informants with different backgrounds participated.

### Data collection

The participatory design process took place at the hospital. The first author (THM) prepared a generative toolkit (Picture 1), in addition, the participants had access to a wide range of other remedies such as paper and cardboard in many colors.

The material was presented at the beginning of the participatory design process and included a short statement about the ambition of the process, which was also stated in the invitation. In addition, the informants were informed verbally and in writing about the study’s details and asking them to sign a consent form highlightning that participating was voluntary and anonymous and that their participation would have no influence on their subsequent treatment as well as explaining that the purpose of the research study.

### The participatory design process

Firstly the Informants were welcomed individually and seated in groups with peer participants, e.g. GPs together, nurses together. The agenda was as follows:

1. Welcome.
2. Short outline of the workshop.
3. Presentation of the program.
4. Brainstorm about important aspects of ensuring the right medication at all times.

The task as presented to the participants and visible on PowerPoint during the whole process:

“Your task is to design the perfect tool to ensure you always get the right medication in the right place. Focus on the solutions and functions of the tool. Build the tool with the remedies we have gathered here. The things you decide to add to the solution must have a function corresponding to a need you or others have- how it looks doesn’t matter, but remember the function of the different parts because we will ask you to present your new tool to the larger group.”

5) Presentation of the generative toolkit.
6) Making a “thing” that can ensure the right medication at all times.
7) All groups present their solution to the other groups.
8) Rounding.

The workshop was facilitated by THM and lasted 2 h and 15 min. There was approximately 1 ½ hours for the making process and half an hour for the presentation of the solutions. The participants had access to refreshments during and after the workshop. The atmosphere was good and empathic addressing the participants own everyday problems and at the same time acknowledging other participants’ situations and working conditions during the presentations.

During the participatory design process, the informants undisturbed generated, tested and elaborated on ideas until the presentation of the models. Data was captured during the presentations to the larger group and were recorded and subsequently transcribed in full, coded, and sorted by THM, JS, and CBM.

The analysis of data follows methods often applied in participatory design studies [28, 29, 31]. We applied an inductive approach focusing on the informants’ descriptions, perceptions, understandings, and ideas. We also applied a deductive analytic strategy based on the themes presented by other informants and identified through the literature. The group discussions were analyzed phenomenally, focusing on the informants’ experiences and perceptions [37, 38].

## Results

The participants were asked to design a tool to illustrate how to ensure patients always get the right medication at the right time. Their solutions were diverse. The patients built a health centre (Additional file 2), the chief physicians a health card containing all key information about the patient (Additional file 3), the general practitioners a communication channel to the hospital (Additional file 4), the nurses employed in general a solution ensuring that the same information is available to all health professionals (Additional file 5), the pharmacists designed a combined database and communication channel (Additional file 6) and hospital- and homecare nurses design the good discharge process (Additional file 7). However, the common factor for all solutions was the focus on an overview of the patient’s diagnoses and treatment. During the analysis, the following themes were identified: (1) Overview of medications, side effects, and diagnoses, (2) Sharing knowledge among healthcare professionals, (3) Timely discharge letters, (4) Does the shared medication record and existing communication platforms provide relevant information to the patient or healthcare professional?

### Overview of medications, side effects, and diagnoses

All participants strived for solutions that created an overview. The patients asked for an overview of their medication, side effects, and interactions. The healthcare professionals aimed for an overview of the patient’s diagnoses and elements important for treatment, such as the presence of a pacemaker. This information should be available in a single solution.

*Chief physician: We are affected by the same fatigue as the other groups have expressed, we do not have the information we need, not even from you (general practice ed.) when you send the patients in, then we face fragmented knowledge and we need to collate and update the information. Is it possible to summarise the information using one solution, preferably a solution that the patient has e.g. a chip or something?*

For patients, the most important thing is to get an overview of the medication, the associated diagnosis, and interactions. Therefore, the patients/relative group suggested a healthcare centre to provide answers regarding medications and health issues.

*Patient: Medication is a huge issue. I’m so uneasy about being sent from one hospital to another. Every time you talk to a doctor, you get a new medication. How does the new medication affect the other medications?*

The patients request contact with a physician responsible for an overview:

*Spouse: there are many people who need to know about the medication, how to take medication, how to act if you get the wrong medication because you can also experience adverse drug reactions.*

In this way, the patients request access to a central healthcare information centre with profound knowledge of the patient’s diagnosis and medications, including side effects and interactions with other medications, and responsibility for the patient’s treatment.

The patients built a health centre that collated information, provided an overview of diagnoses and medications, and gave knowledge about side effects and interactions.

*Patient: When you come to this house, you get an answer you can understand. When you are discharged from hospital, you are often left with new medications, and you are left to your own devices or you have to contact your GP. We request closer cooperation between the hospital and the general practitioners or health care centres. Because sometimes, when you come home, you realise it is difficult to understand the mixed medication you have been given.*

Thus, patients ask for a solution that collates information about diagnoses, medications, and interactions and can explain it to the patients. However, it is a prerequisite for healthcare professionals to be able to create an overview of diagnoses and treatments.

### Sharing knowledge among healthcare professionals

All participating healthcare professionals asked for additional information from other parts of the healthcare sector. All of them have access to SMR, showing the patients’ current prescriptions and medications prescribed within the last ten years, giving profound information regarding the patient’s medication.

The participating homecare and hospital-employed nurses build an illustration of the good discharge illustrating their principal wishes:

*Hospital-employed nurse: We have looked into the available communication tools to see how they can ensure that the medication and the medication management are handled in the best way. […] We have tried to illustrate the path to a good discharge. And the cornerstones […] were that the SMR is updated and were the patient given a sufficient amount of medication to take hom*e *until the new medication could be retrieved or delivered from the pharmacy, […] and that there are prescriptions for the new and previous medication […], and then; who collects it (at the pharmacy ed.) […] - we have our treatment and care plans, we can send them out to each other, but (the homecare nurses ed.): It’s fine that you (the physician ed.) prescribe a new medication, but we also need to know the indication/purpose…*.

As the quote shows, there are many aspects regarding a good discharge. An important part is that the SMR is updated, ensuring primary healthcare the relevant and updated information regarding the medication. It is also important to ensure that the patient has the right and sufficient amount of medication at home and if not, a plan to ensure how the patient can access more or new medication, as well as a plan for a follow-up consultation when needed. Finally, they request information about the diagnoses leading to a new prescription.

### Timely discharge summaries

The GP receives a discharge summary from the hospital when a patient is discharged. However, the GPs also requested more information such as diagnosis, what information was given to the patients, and timely discharge summaries:

*GP: What we lack in this communication channel is that the discharge summay arrives on time and contains the necessary information. If there have been changes in medication, we need to know why. […] The medication that may have been prescribed; is the patient informed well enough about it? […] If they receive dose dispensing, […] then we must also have a home nurse over so that we can get them dosed up as a supplement to their usual medication.*

The GPs ask for different types of information, including that the discharge letters are received quickly. However, this can be logistically difficult for hospital doctors as hospital secretaries are given three days to prepare discharge letters.

The participatory design process shows that the discharge letters are important for the GPs and that it is important that they are received shortly after discharge so that they can contribute to ensuring that the patient always receives the right medication at the right time.

*4) Does the shared medication record and existing communication platforms provide relevant information to the patient or healthcare professional?*

As described above, SMR contains all medication prescribed to the patient within the last ten years. The diagnosis is stated in the discharge letter, although the citations below indicate they don’t always fully meet the wishes of the GPs.

*Chief physician: Do you receive discharge letters that you find informative and make you feel well-prepared (for resuming the treatment of the patient ed)?*

*GP: The problem is if they are the standardized ones, then there will be far too much unnecessary information, and then we will go straight to the conclusion. And then, unfortunately, you may sometimes overlook some important information.*

Thus, too little but also too much irrelevant information can be problematic. The challenge with too much irrelevant information is that the general practitioner cannot form a quick overview of the patient’s treatment at the hospital. Likewise, the chief physician does not want to provide too much information. As a chief physician said when presenting their model:

*Chief physician: That is also why we propose… you have to define what is common because there is no reason for us to know everything that happens out there, because it will not be relevant and focused, and it will require too much sorting work. But there are some common things of mutual benefit that we all should all know.*

In summary, all participating groups request targeted information. They did not request the same information showing that some information should be available to all the participating groups while other information should target specific groups. In this way, a solution/tool to ensure that the patient always gets the right medication should collect the relevant information to allow an overview, and ensure targeted information to the relevant actors to prevent information overload and loss for the healthcare professionals, but also avoid insecurity and confusion for the patients.

## Discussion

The participating healthcare professionals requested targeted information corresponding to the patient’s preferences and expectations. The patients requested one integrated service or a healthcare professional who has the overview of the patient’s diagnosis and medications, including side effects and interactions with other medications, and responsibility for the patient’s treatment. This could be a physician, clinical pharmacist at the hospital, or GP. This corresponds with a systematic review of interventions to increase medication adherence showing that verbal and verbal/written information was the most effective [39]. This study adds that even though the different stakeholders ask for different information, this different information can be contained in one shared tool to be developed ensuring useful and targeted information to all stakeholder groups.

All the informants want a better overview of the patient’s treatment, medication, and diagnoses despite the fact that that medications prescribed to patients are already accessible online to all groups of informants in SMR [24–26] and that GPs already receive a discharge summary from the hospital with suggested follow-up. If needed, the municipalities homecare, receive a patient treatment- and care plan from the hospital typically including information regarding the hospitalization, diagnoses, medication, and required nursing and homecare support after discharge [27]. In summary, all groups already have a large degree of access to information. To ensure the right medication at the right time these data need to be targeted and presented in a way that makes it easy to ensure the patient the right medication at the right time, targeting the different groups and their responsibilities. Hence it may be more important to be able to provide the right information to different groups at the right time, rather than synthesizing the results at this point and, risking not addressing some of the issues presented in further PD processes. Although the participatory design process was about how to ensure that the patient always gets the right medication, the process showed that the stakeholders also want to know and share other related health information, including the diagnosis / medical indication for medication, also described elsewhere [40, 41] and, in the case of the participating patients, side effects, and interactions, aspects also described elsewhere [23, 42, 43]. The GPs also requested what information was given to the patients, as described elsewhere [40], which also should be in a language understandable to the patient, also described elsewhere [17, 44]. The different groups of participants built solutions related to their tasks and their tools. For example, nurses focused on the perfect patient discharge using tools in place with additional elements for improvement. This example demonstrates the relevance and strength of PD and why it is important to invite many different actors in an iterative PD process [31] before a final solution is fully developed which also will allow other possible aspects and solutions to emerge.

### Perspectives

Despite many years of research in sharing information between healthcare professionals [40, 41, 44–46] and the fact, that it is already possible to gather and share information within the framework of Danish legislation through SMR and the discharge summary to the GPs as well as the patient treatment and care plan to the homecare nurses [27] this study show, that targeted information is still requested by the participants. There is a need to ensure that the information is present, that it is easy to find, and does not disappear in an irrelevant information overload.

These findings can strengthen the focus on cross-sectoral communication when combined with other available experiences such as further studies on the use and shortcomings of discharge summaries. The findings can be used for the future process of optimizing existing communication channels between healthcare sectors. Hopefully, the findings can also contribute to developing a “tool” or platform that provides a fast, sufficient, and safe overview for all the health professionals engaged in the individual patient’s care.

The first step towards a solution ensuring that the patient always receives the right medication is to create an overview of what information the different actors want, especially the patients and relatives. This study is the first in a repetitive, iterative PD process to find a solution. The fact that the participants built different solutions shows that different needs can coincide. Therefore, future PD processes must be split between professionals and patients in parallel paths to focus on the professionals’ wishes for an online solution, in combination with a solution that can support citizens’ wishes for a physical location or a possible app that may be of interest among younger and future older generations as described elsewhere [47, 48]. This knowledge can be used to develop a solution during future repeated iterative PD processes developing several prototypes, testing, and developing the common solution gradually [31].

### Strength and limitations

It is a limitation that a relatively small number of different participants attended. However, it is a strength that all central actors are represented; GPs, the hospital, and the municipality as well as some patients and a relative participated in the PD process. We recognize the limited number of patients in our sample, but the Danish society especially among older citizens is quite homogeneous, all have free access to health care, and data was sampled though interviews, so we are not particularly worried about representability among the patient group. In addition PD processes are often conducted with a rather small number of participants, including patients [30, 49, 50] and is known as a reliable method [31, 49, 50].

Bias may occur if the informants do not express their actual attitudes if they feel insecure in the setting. Hence the actors were grouped with like-minded participants to ensure an environment where they did not restrain themselves out of respect for others. All the participants, including the patients and the relative participated and spoke freely, and the atmosphere was friendly and relaxed. The participation of patients and a relative is considered a strength as they enriched the discussion. The first author (THM) is a trained researcher in qualitative methods and ensured that all voices, experiences, and opinions were heard and presented. As a sociologist, THM had no prior knowledge regarding patients’ medications and the problems facing older patients after discharge from the hospital or knowledge of the problems of healthcare professionals.

A further strength of this study is that the participating patients and relatives managed multiple medications daily and were well-functioning. The participants had a high degree of knowledge about their illness and were willing to discuss central issues about managing the disease. Patients are probably the best informants to highlight the factors preoccupying this target group. A further limitation of the study is that frail senior citizens may be underrepresented, and patients taking no particular interest in their medication might be expected to decline participation in the focus group interviews. However, the participation of healthcare professionals enabled the perspectives related to frail patients or patients with no particular interest in their medication to be included.

## Conclusion

All participants in this study state that they lack an overview of patient-related information. Patients lack an overview of their medication, side effects, and interactions. Health professionals lack an overview of the patient’s diagnoses and other factors of importance for the treatment. While the patients wish that the service are available in one physical location, the healthcare professionals wish that important information is gathered, sorted, and accessible to the relevant healthcare professionals online at all times. These two wishes are not mutually exclusive, but important elements should be elaborated upon in future PD processes to ensure that older patients receive the right medication at the right time.

### Electronic supplementary material

Below is the link to the electronic supplementary material.

Supplementary Material 1

Supplementary Material 2

Supplementary Material 3

Supplementary Material 4

Supplementary Material 5

Supplementary Material 6

Supplementary Material 7

Supplementary Material 8

## Data Availability

The datasets are not publicly available due to regulations from The Danish Data Protection Agency.

## Abbreviations

GP: General practitioner
OUH: Odense University Hospital
PD: Participatory design
SMR: an online Shared Medication Record that can be accessed by the patient and healthcare professionals across sectors. In Danish called Fælles Medicinkort (FMK)
