# Supplementary figures and images for: Designing a tool ensuring older patients the right medication at the right time after discharge from hospital– the first step in a participatory design process
Source: BMC Health Serv Res. 2024 Apr 24;24:511. doi: 10.1186/s12913-024-10992-3 (PMC11040918; doi:10.1186/s12913-024-10992-3)

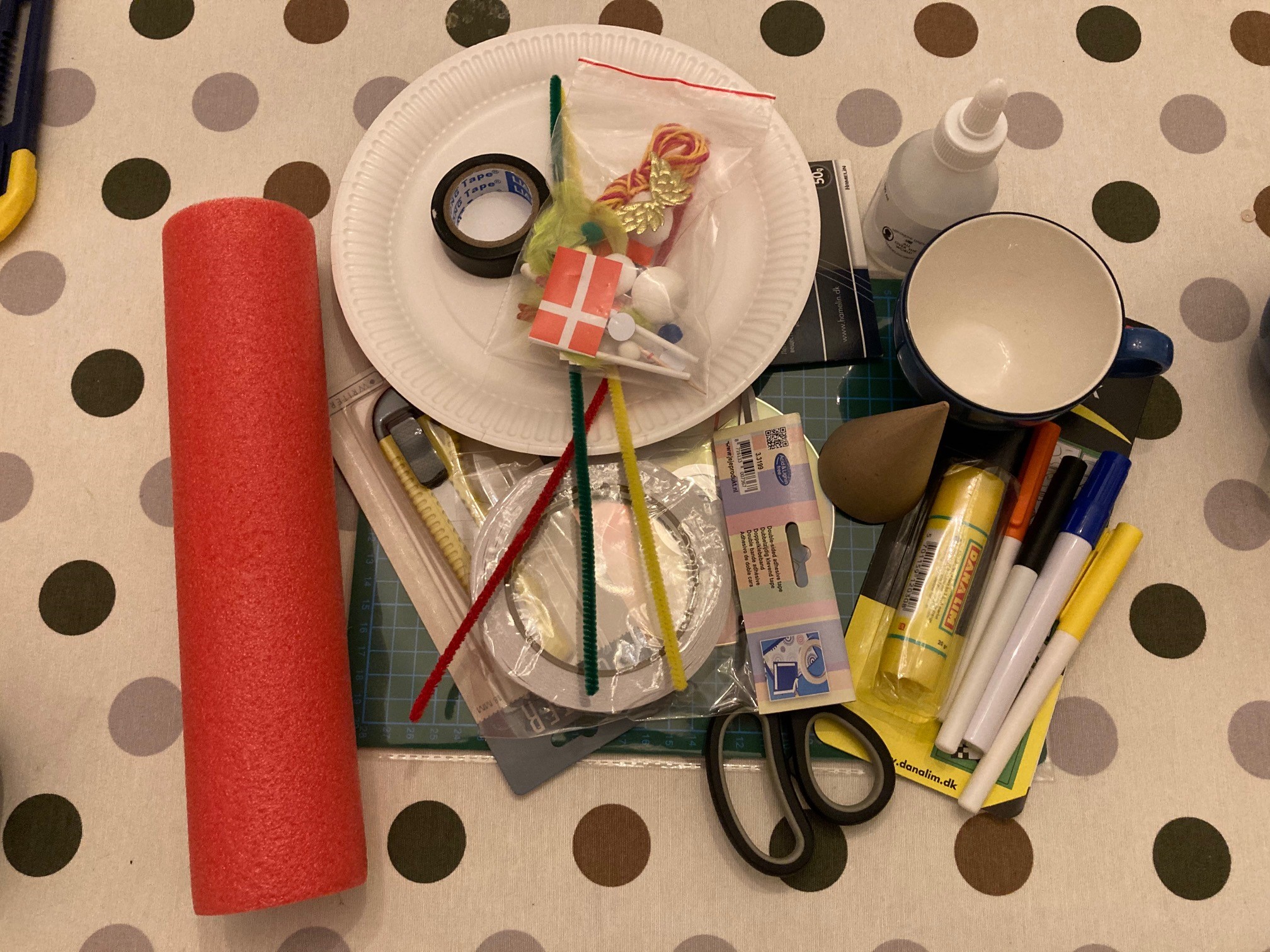

Supplement: Supplementary file 1 — Supplementary Material 1 [file 12913_2024_10992_MOESM1_ESM.jpg]

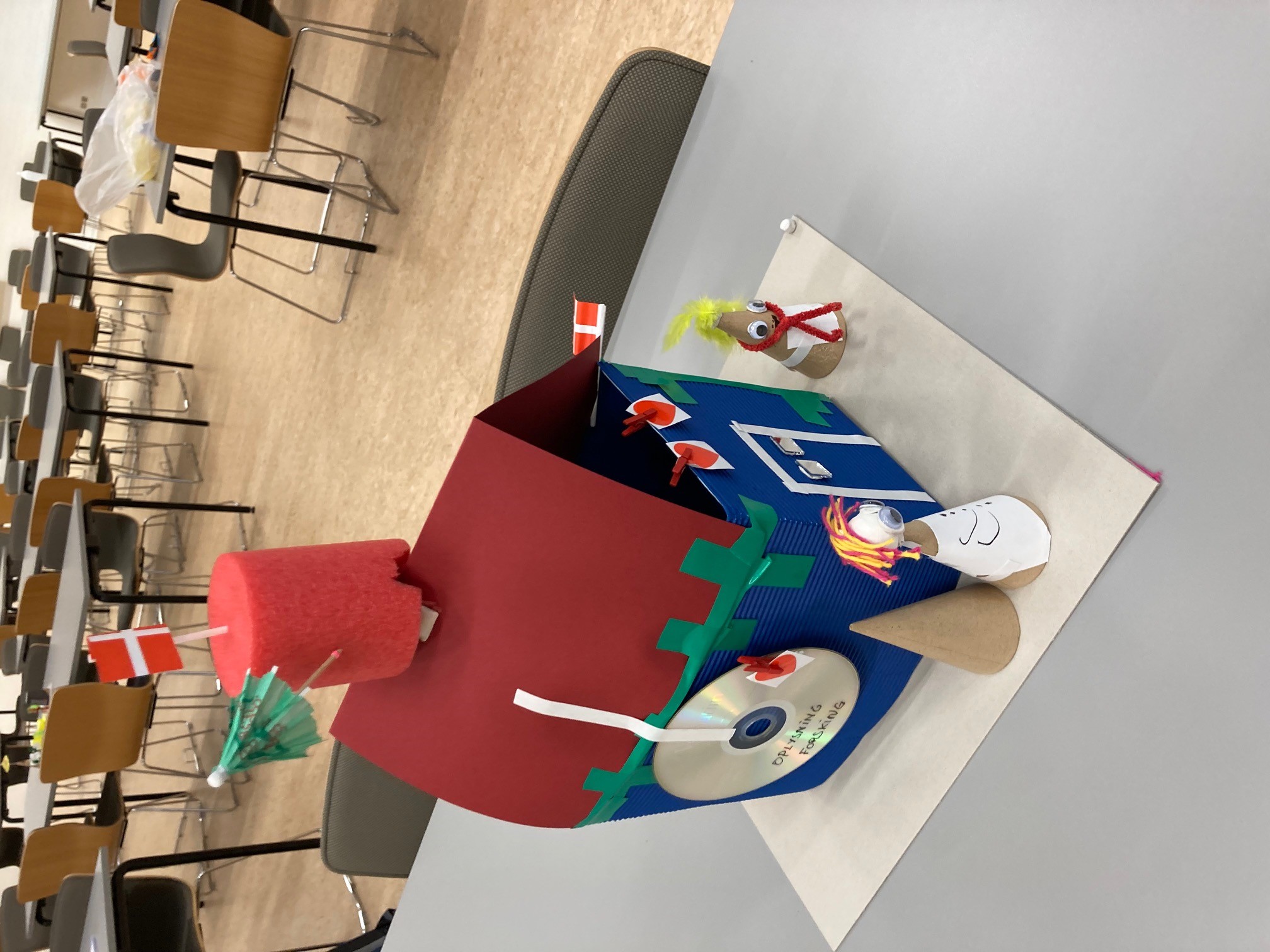

Supplement: Supplementary file 2 — Supplementary Material 2 [file 12913_2024_10992_MOESM2_ESM.jpg]

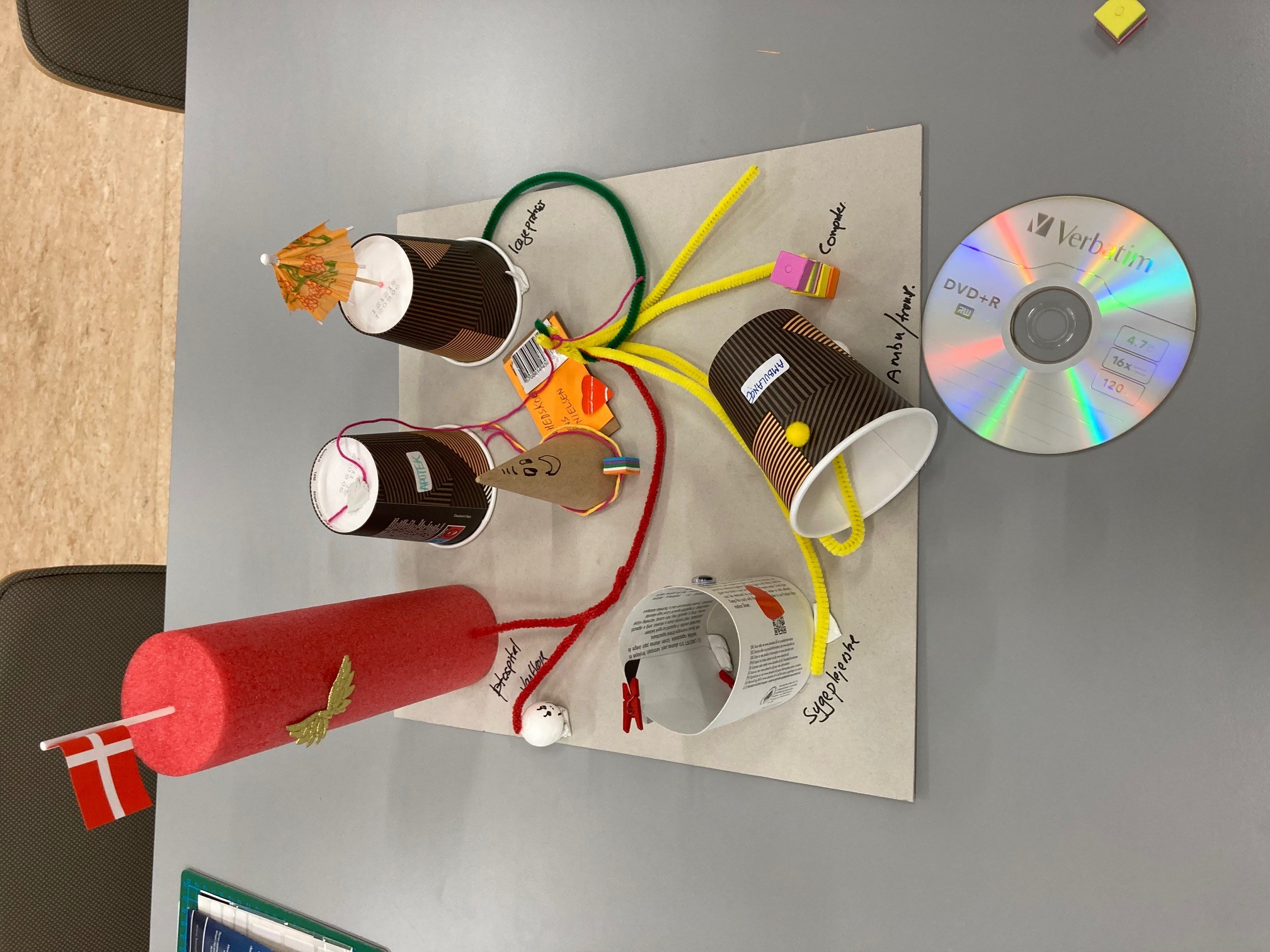

Supplement: Supplementary file 3 — Supplementary Material 3 [file 12913_2024_10992_MOESM3_ESM.jpg]

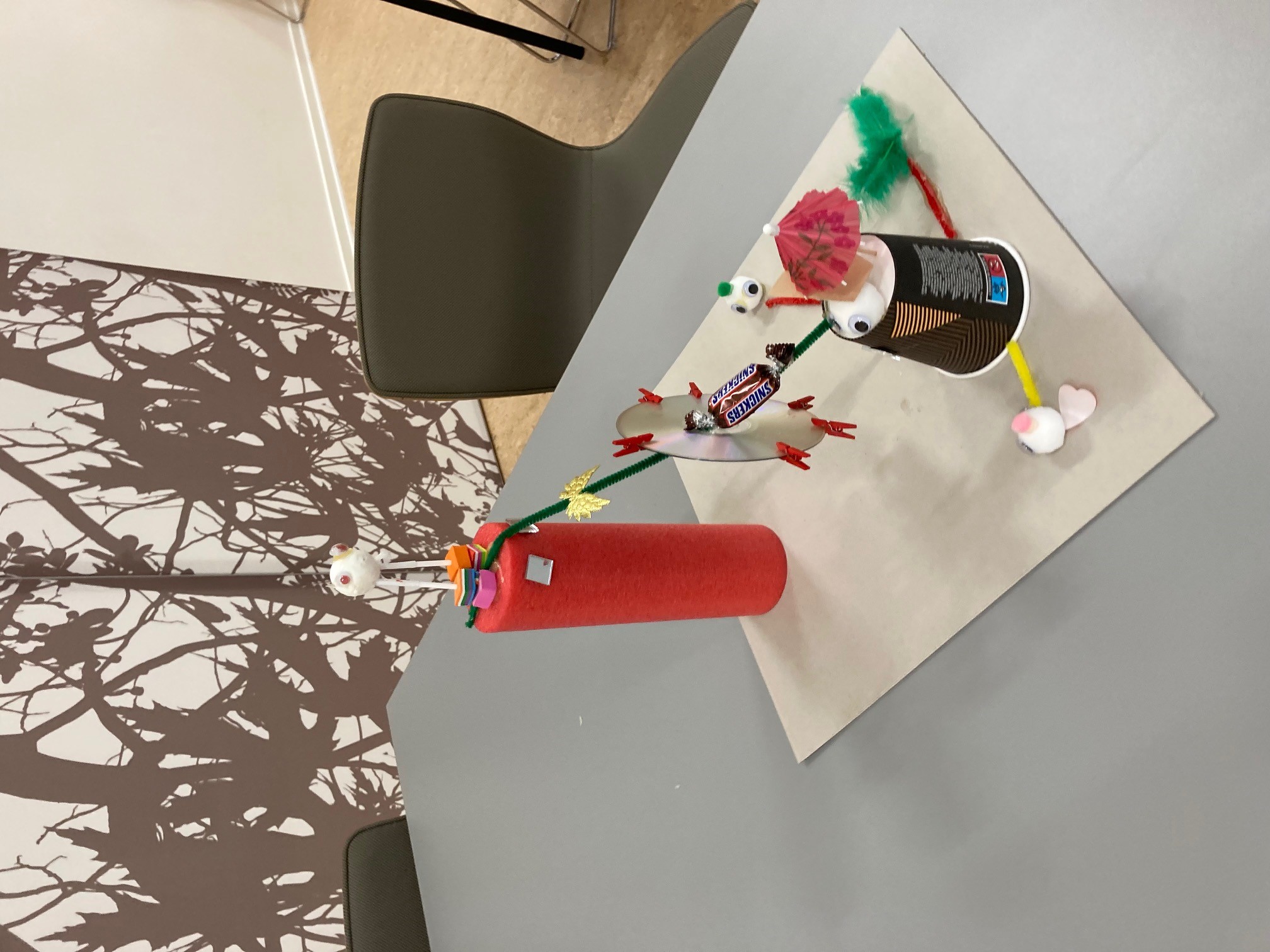

Supplement: Supplementary file 4 — Supplementary Material 4 [file 12913_2024_10992_MOESM4_ESM.jpg]

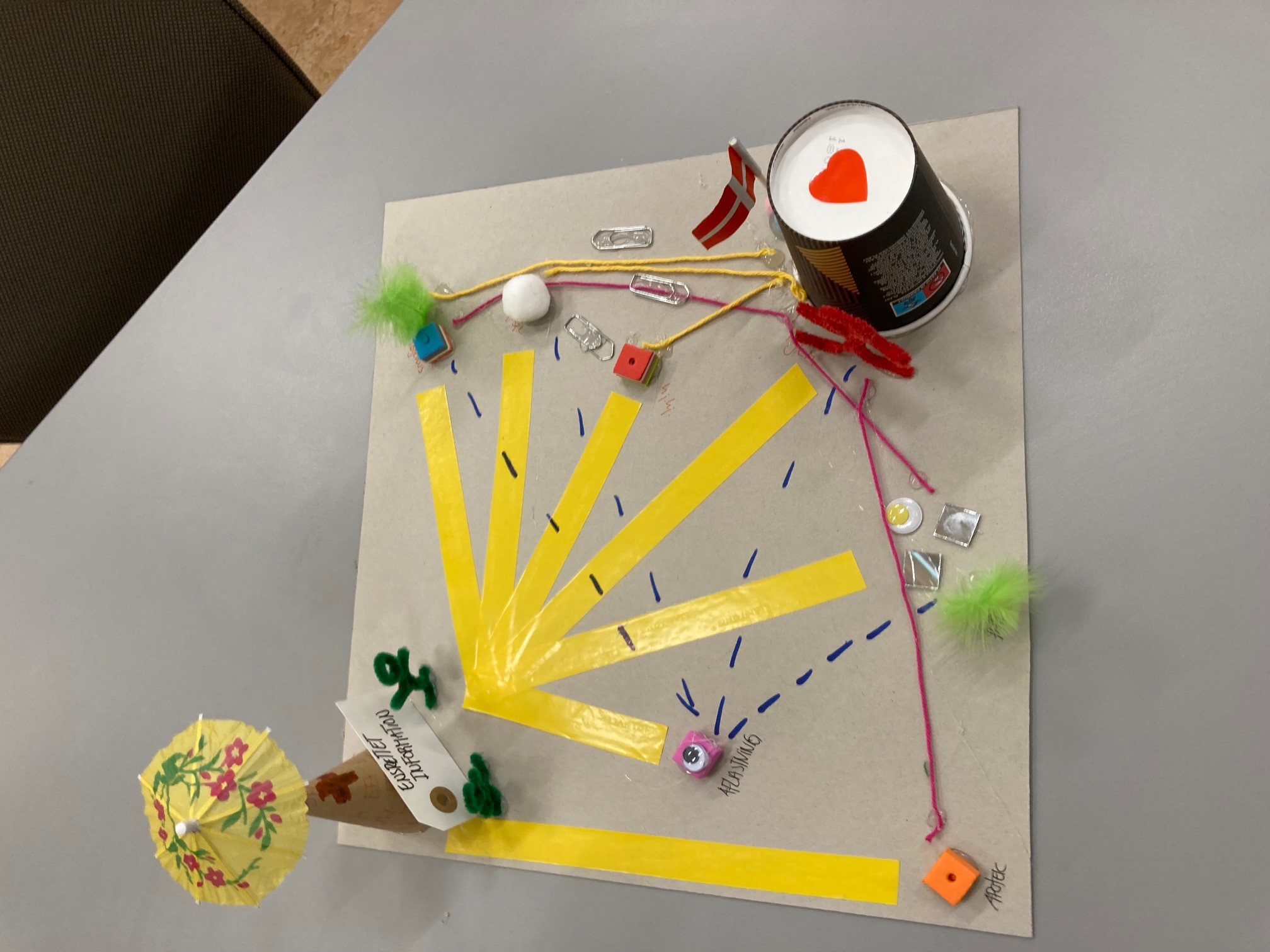

Supplement: Supplementary file 5 — Supplementary Material 5 [file 12913_2024_10992_MOESM5_ESM.jpg]

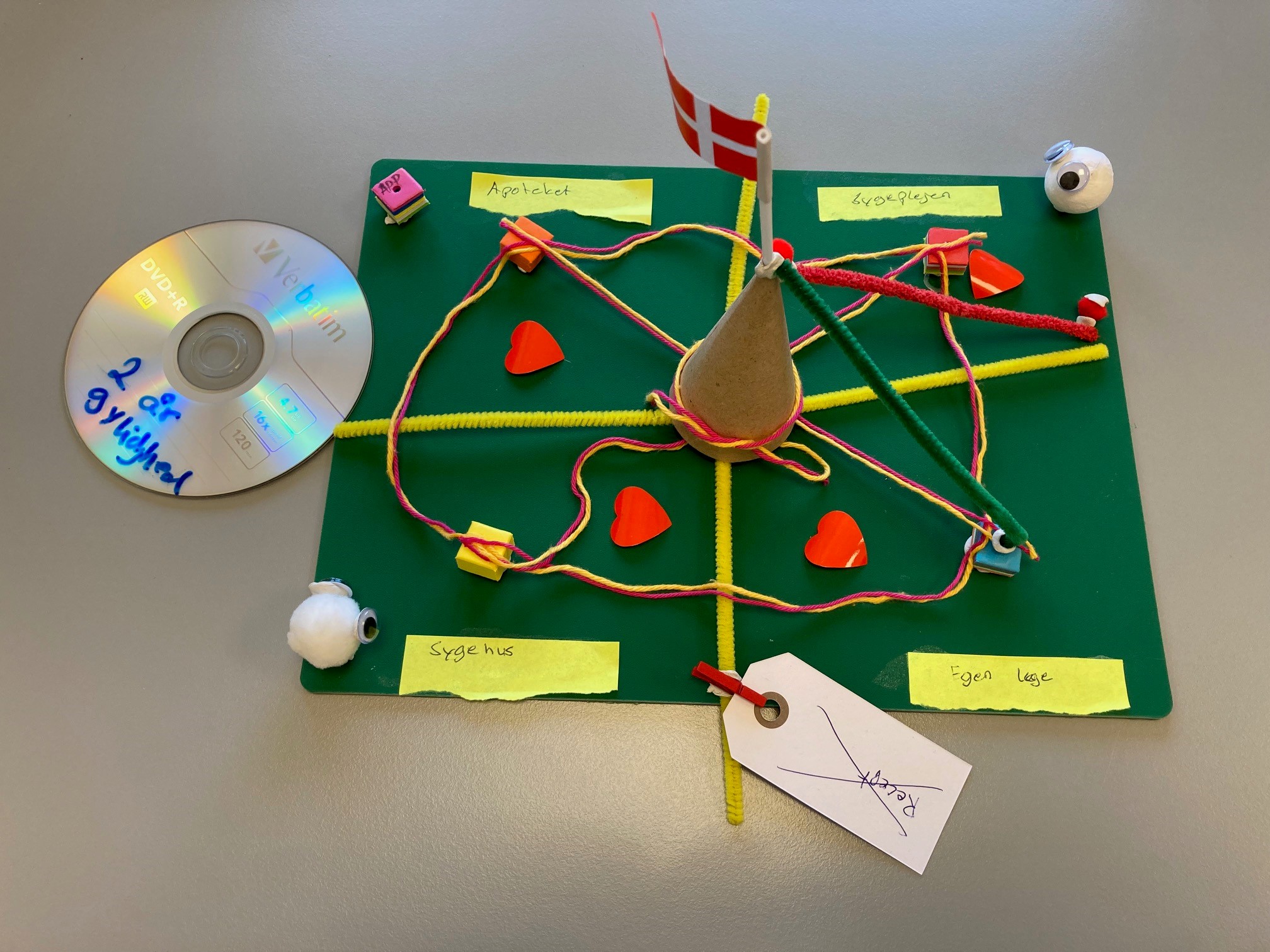

Supplement: Supplementary file 6 — Supplementary Material 6 [file 12913_2024_10992_MOESM6_ESM.jpg]

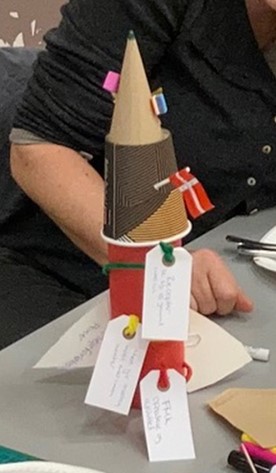

Supplement: Supplementary file 7 — Supplementary Material 7 [file 12913_2024_10992_MOESM7_ESM.jpg]
