# Supplementary material for: Designing a tool ensuring older patients the right medication at the right time after discharge from hospital– the first step in a participatory design process
Source: BMC Health Serv Res. 2024 Apr 24;24:511. doi: 10.1186/s12913-024-10992-3 (PMC11040918; doi:10.1186/s12913-024-10992-3)
Supplement: Supplementary file 8 — Supplementary Material 8 [file 12913_2024_10992_MOESM8_ESM.docx]

**Additional files**

Additional file 1:

- JPG
- The generative toolkit
- A picture showing the generative toolkit was handed out to the participants, in addition, the participants had access to a wide range of other remedies such as paper and cardboard in many colors

Additional file 2:

- JPG
- The Patients built a health centre
- A picture showing the solution created by the patients who built a health centre to provide answers regarding medications and health issues

Additional file 3:

- JPG
- The chief physicians designed a health card
- A picture showing the solution created by the chief physicians who designed a health card containing all key information about the patient

Additional file 4:

- JPG
- The general practitioners designed a communication channel to the hospital
- A picture showing the solution created by the general practitioners who designed a communication channel to the hospital

Additional file 5:

- JPG
- The nurses employed in general practice designed a solution ensuring that the same information is available to all health professionals
- A picture showing the solution created by the nurses employed in general practice who designed a solution ensuring that the same information is available to all health professionals

Additional file 6:

- JPG
- The pharmacists designed a combined database and communication channel
- A picture showing the solution created by the pharmacists who designed a combined database and communication channel

Additional file 7

- JPG
- Hospital and homecare nurses designed the good discharge process
- A picture showing the solution created by the homecare nurses who designed the good discharge process
